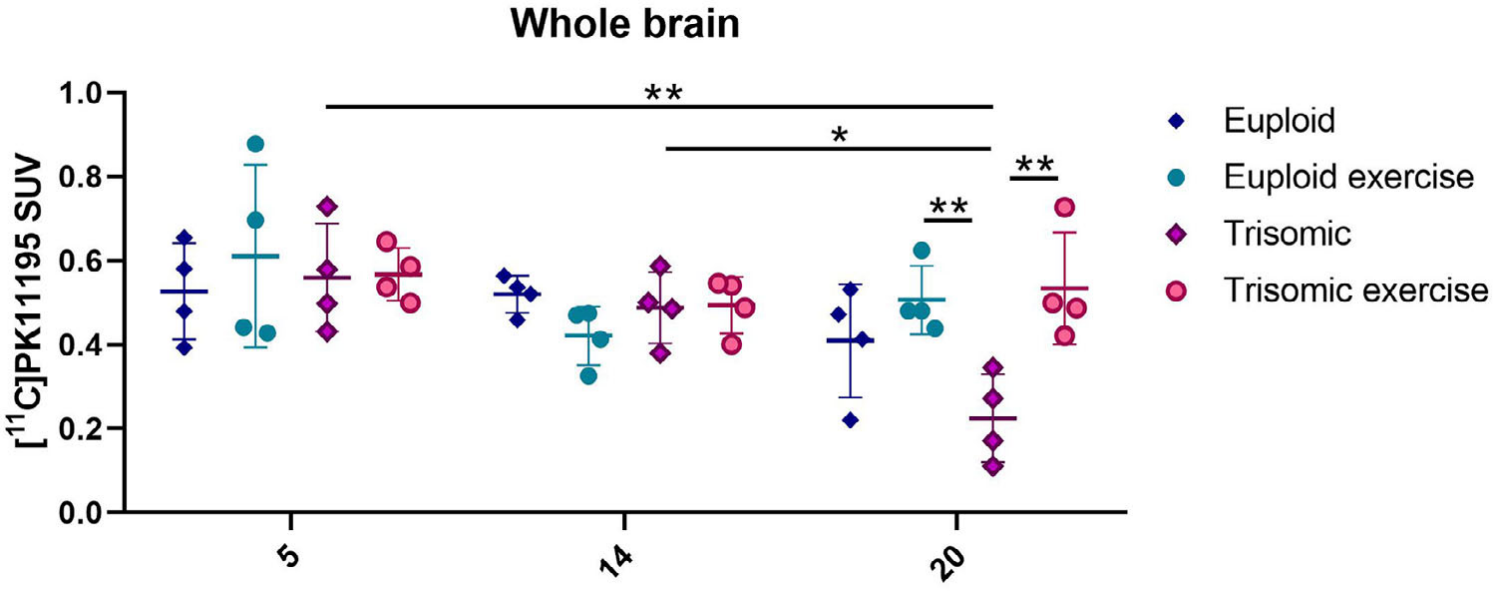# Physical exercise prevents age‐related changes in [^11^C]PK11195 uptake in a mouse model of Down Syndrome

**DOI:** 10.1002/alz.090740

**Published:** 2025-01-09

**Authors:** Chiara Maria Righini, Larissa Estessi de Souza, Jean Marques Brizola, Lidia Emmanuela Wiazowski Spelta, Daniele de Paula de Paula Faria

**Affiliations:** ^1^ University of São Paulo Medical School, São Paulo, São Paulo Brazil

## Abstract

**Background:**

Down syndrome (DS) is associated with mitochondrial dysfunction leading to higher levels of oxidative stress and cell degeneration. This fact, together with the overexpression of AD‐related genes in trisomy 21, increases the risk of developing Alzheimer's disease (AD). Thus, it is important to look for interventions that could prevent mitochondrial damage before symptoms occur. Physical activity is a non‐pharmacological intervention that provides an anti‐inflammatory response and increases the levels of neurotrophic factors that are beneficial for brain health. Therefore, the aim of this study is to evaluate the effect of physical activity on microglial function during the aging process in a transgenic mouse model of DS using [^11^C]PK11195 positron emission tomography.

**Method:**

Ts65Dn male and female mice (euploid and trisomic) were used in this study (CEUA 1292/2019). Animals were subjected to physical activity on a treadmill from 2 to 20 months of age, 3 times per week. [^11^C]PK11195 PET images were acquired at 5, 14, and 20 months of age. For image acquisition, animals were injected intravenously with [^11^C]PK11195. Images were acquired in a small animal PET scanner 30 min after tracer injection, and acquisition took 20 min, with animals kept under anesthesia throughout the acquisition. PET images were co‐registered to a T2‐MRI template and the standardized uptake value (SUV) was calculated in the whole brain.

**Result:**

Throughout the aging process, a decrease in the [^11^C]PK11195 uptake was observed in the trisomic animals at 20 months of age, when compared to the age of 5 months (‐60.7%, 0.56±0.13 vs. 0.22±0.1, p<0.05); and 14 months (‐55.1%, 0.49±0.09 vs. 0.22±0.1, p<0.01). However, the exercised trisomic animals did not present a decrease in the [^11^C]PK11195 uptake at 20 months being comparable (no statistical differences) to the euploid animals (exercise and non‐exercise groups).

**Conclusion:**

Our data suggest that physical activity could prevent a possible deleterious effect of accelerated aging in mitochondrial function, in the presence of the trisomy 21, since lower [^11^C]PK11195 uptake could be associated with mitochondrial dysfunction and reduced expression of the TSPO protein.